# Dabigatran Level Before Reversal Can Predict Hemostatic Effectiveness of Idarucizumab in a Real-World Setting

**DOI:** 10.3389/fmed.2020.599626

**Published:** 2020-12-16

**Authors:** Nicolas Gendron, Richard Chocron, Paul Billoir, Julien Brunier, Laurence Camoin-Jau, Marie Tuffigo, Dorothée Faille, Dorian Teissandier, Juliette Gay, Emmanuelle de Raucourt, Ludovic Suner, Corentin Bonnet, Anne-Céline Martin, Dominique Lasne, Chayma Ladhari, Aurélien Lebreton, Laurent Bertoletti, Nadine Ajzenberg, Pascale Gaussem, Pierre-Emmanuel Morange, Véronique Le Cam Duchez, Alain Viallon, Pierre-Marie Roy, Agnès Lillo-le Louët, David M. Smadja

**Affiliations:** ^1^Université de Paris, Innovative Therapies in Haemostasis, INSERM, Paris, France; ^2^Hematology Department and Biosurgical Research Lab (Carpentier Foundation), AH-HP, Georges Pompidou European Hospital, Paris, France; ^3^Université de Paris, PARCC, INSERM, Paris, France; ^4^Emergency Department, AH-HP, Georges Pompidou European Hospital, Paris, France; ^5^Normandie Univ, UNIROUEN, INSERM Rouen University Hospital, Vascular Hemostasis Unit, Rouen, France; ^6^CHU-Pellegrin, Laboratory of Hematology, Bordeaux, France; ^7^AP-HM, CHU Timone, Laboratory of Hematology, Marseille, France; ^8^CHU Angers, Laboratory of Hematology, Angers, France; ^9^Université de Paris, Laboratory of Vascular Translational Science, INSERM, Paris, France; ^10^Laboratory of Hematology, AH-HP, Bichat Hospital, Paris, France; ^11^CHU Clermont-Ferrand, Emergency Medicine Department, Clermont-Ferrand, France; ^12^Hematology Department, AH-HP, Georges Pompidou European Hospital, Paris, France; ^13^Université de Paris, LVTS, INSERM, Paris, France; ^14^Hematology Department, AP-HP, Hôpital Beaujon, Clichy, France; ^15^Sorbonne Université, Inserm, Centre de Recherche Saint-Antoine, AP-HP, Hôpital Saint-Antoine, Hématologie Biologique, Paris, France; ^16^CHU Sud Réunion, Anaestesiology Department, Saint-Pierre, La Réunion, France; ^17^Cardiology Department, AH-HP, Georges Pompidou European Hospital, Paris, France; ^18^AP-HP, CHU Necker-Enfants Malades, Department of Biogical Hematology, Paris, France; ^19^CHU Montpellier, Centre Régional de Pharmacovigilance, Montpellier, France; ^20^CHU Clermont-Ferrand, Laboratory of Hematology, Clermont-Ferrand, France; ^21^Service de Médecine Vasculaire et Thérapeutique, CHU de Saint-Étienne, INSERM, Université Jean-Monnet, INSERM, CHU de Saint-Étienne, Saint-Étienne, France; ^22^F-CRIN INNOVTE, Saint-Étienne, France; ^23^C2VN, Aix Marseille Univ, INSERM, INRAE, C2VN, Marseille, France; ^24^CHU Saint-Étienne, Emergency Department, Saint-Étienne, France; ^25^CHU Angers, Emergency Department and Vascular Medicine Ward, Université d'Angers, MITOVASC Institut, UMR (CNRS 6015—INSERM 1083), Angers, France; ^26^Département de Pharmacovigilance, AH-HP, Georges Pompidou European Hospital, Paris, France

**Keywords:** idarucizumab, dabigatran, reversal, bleeding, hemostatic effectiveness, rebound, perioperative

## Abstract

**Background:** Idarucizumab has been included in guidelines for the management of bleeding or surgical procedure in dabigatran-treated patients without need for biological monitoring. The aim of the study was to assess the prognostic value of dabigatran plasma level before reversal to test the hemostatic efficacy of idarucizumab. The secondary objectives were (i) to analyze plasma dabigatran level according to the risk of rebound and (ii) to evaluate the incidence of post-reversal non-favorable clinical outcomes (including thromboembolism, bleeding, antithrombotic, and death) and antithrombotic resumption.

**Methods and Results:** This was an observational multicentric cohort study, which included all French patients who required idarucizumab for dabigatran reversal. Between May 2016 and April 2019, 87 patients from 21 French centers were enrolled. Patients received idarucizumab for overt bleeding (*n* = 61), urgent procedures (*n* = 24), or overdose without bleeding (*n* = 2). Among patients with major bleeding (*n* = 57), treatment with idarucizumab was considered effective in 44 (77.2%) of them. Patients who did not achieve effective hemostasis after reversal had a significantly higher mean level of plasma dabigatran at baseline (524.5 ± 386 vs. 252.8 ng/mL ± 235, *p* = 0.033). Furthermore, patients who did not achieve effective hemostasis after reversal had less favorable outcomes during follow-up (46.2 vs. 81.8%, *p* = 0.027). ROC curve identified a cutoff of 264 ng/mL for dabigatran level at admission to be predictive of ineffective hemostasis. No plasma dabigatran rebound was observed after reversal in patients with dabigatran plasma level < 264 ng/mL at baseline.

**Conclusion:** This retrospective study shows that dabigatran level before reversal could predict hemostatic effectiveness and dabigatran plasma rebound after idarucizumab injection.

## Introduction

The specific reversal agent idarucizumab is a humanized monoclonal antibody fragment that binds dabigatran with a very high affinity. The RE-VERSE AD trial (1) showed the efficacy and safety of idarucizumab to reverse the anticoagulant effect of dabigatran within 4 h after its administration in dabigatran-treated patients who experienced serious bleeding or required urgent invasive procedures. Although small case series (2, 3) on the use of idarucizumab have been reported, there are only three large studies in a real-world setting. First, a cohort of 20 hospitals in the Netherlands (4) enrolled 88 patients; among them, 53 had severe bleeding, and 35 required urgent surgical intervention. Effective hemostasis was achieved in two-thirds of bleeding patients and was associated with a lower mortality risk. Clinical outcomes were considered similar to those observed in the RE-VERSE AD trial, regarding recurrent bleeding, thromboembolism, and mortality rate. Second, a large national multicentric retrospective study from the United States (5) included 266 patients exposed to idarucizumab who were compared to 1,345 non-exposed patients. In patients with gastrointestinal bleeding (GIB), there was no difference in terms of in-hospital mortality between patients exposed to idarucizumab and those non-exposed, but in case of intracranial hemorrhage (ICH), an increased risk of mortality was reported among patients exposed to idarucizumab. Third, a German national retrospective study (6) enrolled 120 dabigatran-treated patients who received idarucizumab, 80 of whom with ischemic stroke and 40 with intracranial bleeding (ICH in 27 patients). In patients receiving intravenous thrombolysis following idarucizumab, 78% showed a median improvement on the National Institutes of Health Stroke Scale. Moreover, hematoma growth was observed in 11.1% of patients with ICH.

According to international guidelines, idarucizumab is indicated in dabigatran-treated patients in case of life-threatening and/or uncontrolled bleeding or situations requiring a rapid reversal of dabigatran anticoagulant effects such as urgent surgery. However, no specific dabigatran monitoring is currently recommended either before reversal or during follow-up (7) due to the lack of consistent biological data during clinical trials. However, reappearance or a rebound of dabigatran levels was observed in 23.0% patients in RE-VERSE AD trial (1). Since then, several cases of reversal failure, plasma dabigatran rebound and/or re-bleeding have been published (2, 3, 8, 9). Interestingly, we previously demonstrated that a risk of rebound in circulating dabigatran after reversal could be predicted by an initial dabigatran plasma level above 200 ng/mL. In this context of bleeding or emergency procedures, it is important to note that routine hemostasis testing, such as prothrombin time and activated partial thrombin time, is inappropriate to monitor the dabigatran level (10, 11).

Our study describes retrospective data collected from 21 French centers from May 2016 to April 2019. The aim was to assess the relationship between dabigatran level before reversal with idarucizumab and the evolution of patients—in particular, hemostatic effectiveness in patients who received idarucizumab for major bleeding or who needed urgent procedures. This study also assessed the prognostic value of dabigatran plasma level at admission to evaluate the risk of rebound and clinical outcomes.

## Materials and Methods

### Study Design and Setting

This observational retrospective cohort study included all French patients who had received idarucizumab for dabigatran reversal and who were reported to the French pharmacovigilance network. Data from patients were retrospectively collected from institutions by a nationwide survey conducted by the French pharmacovigilance centers from May 2016 to April 2019. No exclusion criteria were applied. The study was performed in accordance with the Declaration of Helsinki. The institutional review board of each center approved the study, and anonymous data collection was declared to the appropriate authorities (authorization protocol number: CNIL-1922081).

### Data Collection

Baseline characteristics of patients (demographic, clinical, cardiovascular risk factors, CHA_2_DS_2_-VASc score, and body mass index), and surgical and biological data were retrieved from the pharmacovigilance reports and the medical records. Indication for the use of idarucizumab was recorded: life-threatening or uncontrolled bleeding requiring medical intervention, emergency surgery, or invasive procedure, and any other setting including dabigatran overdose. Additional blood products and/or pro-hemostatic agents used during reversal, patient's outcomes, and antithrombotic resumption data were also retrieved from the medical records. Creatinine clearance (CrCl) was calculated using the Cockcroft and Gault formula.

### Study Outcomes

The primary objective was to assess the prognostic value of dabigatran plasma level tested before reversal with idarucizumab to evaluate the hemostatic effectiveness in major bleeding patients.

The secondary objectives were (i) to analyze plasma dabigatran level according to the risk of rebound to replicate our previous study (3). Rebound was defined as an increase in the plasma concentration above the 30 ng/mL threshold of detection after reversal and (ii) to evaluate the incidence of non-favorable clinical outcomes including symptomatic thromboembolism, re-bleeding or non-cessation of the bleeding, allergic reaction, and deaths at 5, 30, and 90 days after dabigatran reversal. Furthermore, antithrombotic resumption after reversal and timing for resuming antithrombotic was also reported.

### Definition of Dabigatran-Related Bleeding

Information regarding the location of bleeding events (GIB, ICH, or other defined locations) was collected. All bleeding dabigatran-related events that needed idarucizumab injection were reviewed retrospectively by two hematologists (NG and DMS) and classified according to the criteria of the International Society on Thrombosis and Haemostasis (ISTH) (12). In short, major bleeding was defined as fatal bleeding or symptomatic bleeding in a critical area or organ or bleeding causing a hemoglobin level of 20 g/L or more or leading to transfusion of >2 U of packed red blood cells (PRBCs). Since all patients included in this study were hospitalized to receive idarucizumab, non-major bleeding fulfilled non-major clinically relevant criteria (13) defined as any sign or symptom of hemorrhage that did not meet the criteria for the ISTH definition of major bleeding, but did meet at least one of the following criteria: (i) requiring medical intervention by a healthcare professional; (ii) leading to hospitalization or increased level of care; or (iii) prompting a face-to-face evaluation.

Effective clinical hemostasis after treatment of a major bleeding using idarucizumab was reviewed and classified as binary outcome, i.e., effective or ineffective according to the ISTH criteria (14). For this assessment, medical records were screened for hemoglobin level, transfusion, invasive procedures reports, and diagnostic imaging reports.

### Statistical Analysis

#### Descriptive Analysis

Continuous data were expressed as mean ± standard deviation (SD). Categorical data were expressed in numbers (*n*) and percentages. We compared patient and biological characteristics at admission after dabigatran reversal according to idarucizumab indication (bleeding, urgent procedure, and other), to hemostatic effectiveness, to initial plasma dabigatran level (≥264 ng/mL) and to dabigatran rebound, using Fisher's exact test for categorical variables and Mann–Whitney–Wilcoxon test for continuous variables (15, 16). We also compared patient characteristics and outcomes after dabigatran reversal according to renal function using the Cochran–Armitage test for trend for categorical variables and the Kruskal–Wallis test for continuous variables (17).

#### Assessment of the Initial Dabigatran Plasma Level as a Biomarker of Hemostatic Efficacy

We generated a receiver operating characteristic (ROC) curve (18, 19) to assess the prognostic value of plasma dabigatran level before reversal in regard to the hemostatic efficacy of idarucizumab and to find an appropriate cutoff point of dabigatran plasma measurement as biomarker. We calculated the area under the curve (AUC).

We used the Kaplan Meier survival curve and the log rank test to compare time with plasma dabigatran rebound and overall survival after reversal according to identified dabigatran cutoff before reversal (20, 21).

We used logistic regression to determine whether the level of dabigatran (as a categorical dependent variable dichotomized according to the cutoff of 264 ng/mL) adjusted on the CrCl (as a categorical dependent variable dichotomized according to the cutoff of 30 mL/min) was associated with the hemostatic efficacy (22).

All analyses were two-sided, and a *p*-value lower than 0.05 was considered to be significant. Statistical analysis was performed using R studio software [RStudio Team (2015). RStudio: Integrated Development for R. RStudio, Inc., Boston, MA, USA].

## Results

### Characteristics of Study Subjects

From May 2016 to April 2019, 21 French centers enrolled 87 patients who received 92 injections of idarucizumab for dabigatran reversal. The demographic, clinical, and biological characteristics of patients are reported in Table 1. Briefly, the mean age of patients was 79.7 years, and 62.1% were males. Mean CrCl at admission was 50.5 mL/min. Almost all patients were treated with dabigatran for non-valvular atrial fibrillation (95.4%), of whom 60.9% received the 110mg twice-daily dose. Idarucizumab injections were administered to 61 (70.1%) patients for overt bleeding, in 24 (27.6%) patients for various urgent procedures, and in two (2.3%) for dabigatran overdose without bleeding. Outcomes of patients from these three patient groups are detailed in Supplementary Tables 1–3. Most events in the bleeding group were GIB (50.8%), ICH (34.2%), and ilio-psoas hematoma (4.9%). Urgent procedures were varied and are reported in Supplementary Table 2. All patients were initially given the full dose of two vials (2 × 2.5 g) of idarucizumab, whereas four (4.6%) patients received a second dose, and only one received a third injection (Supplementary Table 4).

**Table 1:** Patient characteristics at admission and outcomes after dabigatran reversal.

**Patient characteristics**	**Bleeding (*N* = 61)**	**Urgent procedure (*N* = 24)**	Others^*^ (*N* = 2)	**All patients (*N* = 87)**
Age–year–mean (±SD)	80.8 (±11.0)	77.2 (±16.0)	75.5 (±4.9)	79.7 (±12.5)
Male sex—*n* (%)	37 (60.7)	17 (70.8)	0 (0.0)	54 (62.1)
CHA_2_DS_2_-VASc score–mean (±SD)	3.12 (±1.6)	3.83 (±1.4)	4.50 (±0.7)	3.32 (±1.5)
BMI–kg/m^2^-mean (±SD)	25.6 (±4.1)	26.9 (±4.3)	30.6 (±6.2)	26.2 (±4.3)
CrCl–mL/min–mean (±SD)	50.8 (±25.6)	53.9 (±24.2)	7.9 (±4.1)	50.5 (±25.1)
Hemoglobin g/L–mean (±SD)	108 (±34)	129 (±30)	100 (±28)	114 (±34)
**Dose of dabigatran—*****n*** **(%)**				
150 mg twice daily	16 (26.2)	10 (41.7)	2 (100.0)	28 (32.2)
110 mg twice daily	40 (65.6)	13 (54.2)	0 (0.0)	53 (60.9)
75 mg twice daily	3 (4.9)	0 (0.0)	0 (0.0)	3 (3.4)
Missing data	2 (3.3)	1 (4.1)	0 (0.0)	3 (3.4)
**Indication for dabigatran—*****n*** **(%)**				
NVAF	59 (96.7)	22 (91.7)	2 (100.0)	83 (95.4)
VTE	1 (1.6)	2 (8.3)	0 (0.0)	3 (3.4)
Others	1 (1.6)	0 (0.0)	0 (0.0)	1 (1.1)
**Laboratory tests at baseline (before idarucizumab administration)**				
Prolonged aPTT—*n*/evaluable patients (%)	47/54 (87.0)	18/21 (85.7)	2/2 (100.0)	67/77 (87.0)
Prolonged PT—*n*/evaluable patients (%)	41/55 (74.5)	16/21 (76.2)	2/2 (100.0)	59/78 (75.6)
Dabigatran, ng/mL–mean (±SD)	327 (±303)	125 (±134)	1,014–2,881	323 (±477)
≥2 injections of idarucizumab—*n* (%)	2 (3.3)	1 (4.1)	2 (50.0)	5 (5.7)

*Note. BMI, body mass index; CrCl, creatinine clearance; NVAF, non-valvular atrial fibrillation; VTE, venous thromboembolic events; aPTT, activated partial thromboplastin time; PT, thromboplastin time*.

* *These two patients (n°86 and n°87) received an idarucizumab injection for dabigatran overdose without bleeding*.

Blood products and prohemostatic agents were used in 46 (58.2%) of 79 assessable patients (Supplementary Table 5). The most frequently used products were PRBCs. Regarding the bleeding group (*n* = 61), 31 (50.8%) patients received a mean of 1.49 (±1.8) PRBC unit/patient. Otherwise, five (8.2%) patients were given fresh frozen plasma and four (6.6%) prothrombin complex concentrates (PCC). Multiple hemostatic agents were required for 9.8% (6/61) patients. Among patients requiring urgent surgery (*n* = 24), eight (33.3%) received 1.10 (±1.7) unit of PRBC/patient, five (20.8%) patients received fresh frozen plasma and two (8.3%) PCC, and five (20.8%) required multiple hemostatic agents. It should be noted that neither rFVIIa nor activated PCC was used.

After reversal, antithrombotic therapy was resumed in 47 (54.0%) patients, 27 (44.3%) from the bleeding group and 18 (75.0%) from the urgent procedure group (Supplementary Table 6). Dabigatran was resumed in 15 (24.6%) patients in the bleeding group and in 12 (50.0%) patients in the urgent procedure group after a mean time of 11.5 days (±24.5) and 5.1 days (±6.8), respectively. According to the outcomes in the bleeding group, the mean time until antithrombotic resumption was 5.5 days (±4.6) for GIB and 33.4 days (±49.6) for ICH.

Among the study patients, 84 (96.6%) had a laboratory test prior to idarucizumab administration. Specific dabigatran plasma measurement was available for 47 patients (59.4%). Diluted thrombin time was used in most cases (*n* = 44). Three (#37, #38, and #39) were quantified by LC/MS. Among evaluable patients, 79 (94.0%) had abnormal hemostasis tests at baseline including prolonged PT, prolonged aPTT, and/or dabigatran plasma level ≥30 ng/mL.

### Patient Post-Reversal Outcomes

During a mean follow-up of 109 days (±190), 65 patients (74.7%) had a favorable outcome (Supplementary Tables 1–3). Three thrombotic events (3.4%) were reported: one pulmonary embolism, one stroke, and one catheter-related venous thrombosis in a cancer patient. One patient developed an anaphylactic reaction within minutes after idarucizumab injection. Eighteen patients died (20.7%) during hospitalization or follow-up. More specifically, mortality rate at 5, 30, and 90 days was 16.4, 19.7, and 23.0%, respectively, in the bleeding group, and 8.3, 12.4, and 16.7% in the urgent procedure group (Supplementary Table 7).

Causes of death are summarized in Supplementary Table 7. In the bleeding group, there were six (9.8%) fatal bleedings, three (4.9%) severe infections, three (4.9%) multivisceral failures, one (1.6%) cardiac arrest, and one (1.6%) disseminated intravascular coagulation. In the urgent procedure group, there were two (8.3%) fatal bleedings and two (8.3%) multivisceral failures. After reversal, overall survival at 90 days did not significantly differ between the bleeding and the urgent procedure groups (Supplementary Figure 1, 79.3 vs. 83.3%, *p* = 0.92).

### Hemostatic Effectiveness of Idarucizumab

Among the 61 patients admitted for bleeding, 58 (95.1%) were considered as major bleeding events according to the ISTH criteria (12). Dabigatran reversal with idarucizumab was considered effective according to ISTH (14) in 44 out of 57 (77.2%) evaluable patients (Table 2).

**Table 2:** Clinical and biological characteristics at admission of evaluable major bleeding patients with or without hemostatic effectiveness after dabigatran reversal.

**Patient characteristics**	**Hemostatic effectiveness**		
	**Yes (*n* = 44)**	**No (*n* = 13)**	***p*-Value**
Age–year–mean (±SD)	80.2 (±11.5)	83.2 (±10.3)	0.39
Male sex—*n* (%)	28 (63.6)	7 (53.8)	0.75
CHA_2_DS_2_-VASc score–mean (±SD)	3.1 (±1.5)	3.3 (±1.9)	0.71
BMI–kg/m^2^-mean (±SD)	25.5 (±4.2)	26.3 (±4.4)	0.63
CrCl–mL/ min–mean (±SD)	48.5 (±24.5)	58.7 (±29.2)	0.24
Hemoglobin g/L–mean (±SD)	107.0 (±34.1)	112.31 (±34.4)	0.63
Dabigatran–ng/mL–mean (±SD)	252.8 (±235)	524.5 (±386)	**0.033**
Favorable outcome—*n* (%)	36 (81.8)	6 (46.2)	**0.027**
Death—*n* (%)	7 (15.9)	6 (46.2)	**0.056**

*BMI, body mass index; CrCl, creatinine clearance. Bold values are corresponding to statistical differences with p-value < 0.005*.

Patients who did not achieve effective hemostasis after reversal had a significantly higher mean level of dabigatran at admission (524 ± 386 vs. 252 ng/mL ± 235, *p* = 0.033). Second, they experienced less favorable outcomes (46.2 vs. 81.8%, *p* = 0.027) during follow-up, with a trend to more deaths (46.2 vs. 15.9%, *p* = 0.056). When classifying major bleeding patients according to renal function, we found that renal impairment was associated with increased baseline dabigatran level (Table 3), although effective hemostasis did not correlate with CrCl (*p* = 0.23) nor with the distribution of renal function into four groups (*p* for trend test 0.12 and p for Pearson's chi-squared test 0.48).

**Table 3:** Patient characteristics and outcomes after dabigatran reversal according to renal function.

	**Renal function**				
	**Normal ≥80 mL/min**	**Mild 50 to** **<80 mL/min**	**Moderate 30 to** **<50 mL/min**	**Severe <30 mL/min**	***p-*Value**
Patient characteristics—*n* (%)	10 (13.5)	22 (29.7)	26 (35.2)	16 (21.6)	-
Age – year–mean (±SD)	67.9 (±12.9)	73.9 (±15.9)	83.4 (±8.42)	85.9 (±6.7)	** <0.001**
Male sex—*n* (%)	8 (80.0)	19 (86.4)	14 (53.8)	6 (37.5)	**0.008**
CHA_2_DS_2_-VASc score–mean (±SD)	1.6 (±0.5)	3.1 (±1.6)	3.9 (±1.6)	3.6 (±1.1)	**0.003**
BMI–kg/m^2^-mean (±SD)	28.6 (±2.3)	26.9 (±4.9)	24.5 (±3.8)	27.0 (±4.4)	0.13
Plasma creatinine–μmol/L, mean (±SD)	81.0 (±13.3)	92.7 (±14.8)	117.0 (±28.9)	332.8 (±252.9)	** <0.001**
CrCl–mL/ min–mean (±SD)	96.4 (±12.4)	65.5 (±7.6)	39.0 (±5.2)	20.2 (±7.5)	** <0.001**
Hemoglobin g/L–mean (±SD)	139.4 (±26.7)	118.1 (±37.9)	112.8 (±25.9)	84.1 (±30.4)	** <0.001**
Dabigatran–ng/mL–mean (±SD)	109.0 (±117)	142 (±192)	279 (±200)	678 (±811)	**0.031**
**Dose of dabigatran—*****n*** **(%)**					
150 mg BID	2 (20.0)	13 (59.1)	18 (69.2)	10 (62.5)	0.084
110 mg BID	6 (60.0)	8 (36.4)	7 (26.9)	5 (31.2)	0.084
75 mg BID	0 (0.0)	1 (4.5)	0 (0.0)	1 (6.2)	0.084
Missing data	2 (20.0)	0 (0.0)	1 (3.8)	0 (0.0)	0.084
**Idarucizumab indications—*****n*** **(%)**					
Bleeding	8 (80.0)	12 (54.5)	21 (80.8)	10 (62.5)	0.061
GIB	1 (10.0)	6 (27.3)	11 (42.3)	8 (50.0)	**0.049**
ICH	5 (50.0)	5 (22.7)	8 (30.8)	0 (0.0)	**0.049**
Major bleeding	8 (80.0)	11 (50.0)	21 (80.8)	10 (62.5)	0.27
Urgent procedure	2 (20.0)	10 (45.5)	5 (19.2)	4 (25.0)	0.061
Others^*^	0 (0.0)	0 (0.0)	0 (0.0)	2 (12.5)	0.061
**Additional blood products or pro-hemostatic agents**	4 (40.0)	12 (54.5)	16 (61.5)	10 (62.5)	0.58
PRBCs—*n* (%)	0.30 (0.67)	1.79 (2.39)	1.32 (1.21)	2.36 (1.86)	**0.038**
FFP—*n* (%)	0.00 (0.00)	0.70 (1.53)	0.32 (0.89)	0.36 (1.08)	0.41
PCC—*n* (%)	2 (20.0)	2 (9.1)	2 (7.7)	0 (0.0)	0.56
Platelets transfusion—*n* (%)	0 (0.0)	3 (13.6)	1 (3.8)	0 (0.0)	0.37
Tranexamic acid—*n* (%)	0 (0.0)	1 (4.5)	2 (7.7)	0 (0.0)	0.66
Fibrinogen—*n* (%)	10 (100.0)	19 (86.4)	22 (84.6)	14 (87.5)	0.64
Vitamin K—*n* (%)	0 (0.0)	0 (0.0)	1 (3.8)	0 (0.0)	0.55
Missing data—*n* (%)	0 (0.0)	2 (9.1)	3 (11.5)	2 (12.5)	0.58
**Patient outcomes**					
Hemostatic effectiveness/evaluable patients—*n* (%)	5/8 (62.5)	8/12 (66.7)	17/21 (81.0)	8/9 (89)	0.12
Favorable outcomes—*n* (%)	8 (80.0)	13 (59.1)	19 (73.1)	15 (93.8)	0.11
Death—*n* (%)	2 (20.0)	7 (31.8)	5 (19.2)	1 (6.2)	0.29
Antithrombotic resumption—*n* (%)	3 (30.0)	11 (50.0)	13 (50.0)	10 (62.5)	0.69
Dabigatran resumption—*n* (%)	2 (20.0)	6 (27.3)	10 (38.5)	6 (37.5)	0.59
Timing for resuming antithrombotic–days–mean (±SD)	4.0 (±3.0)	5.6 (±4.6)	16.1 (±39.7)	10.0 (±7.5)	0.71

*Characteristic of analyzable patients included in the study according to renal impairment (n = 74)*.

*M, male; F, female; SD, standard deviation; BMI, body mass index; CrCl, creatinine clearance; BID, twice a day; GIB, gastrointestinal bleeding; ICH, intracranial hemorrhage; NA, non-available; PRBCs, packed red blood cells; FFP, fresh frozen plasma; PCC, prothrombin complex concentrate*.

* *Patients #86 and #87 received idarucizumab for dabigatran overdose without bleeding*.

*Bold values are corresponding to statistical differences with p−value < 0.005*.

**Figure 1:**
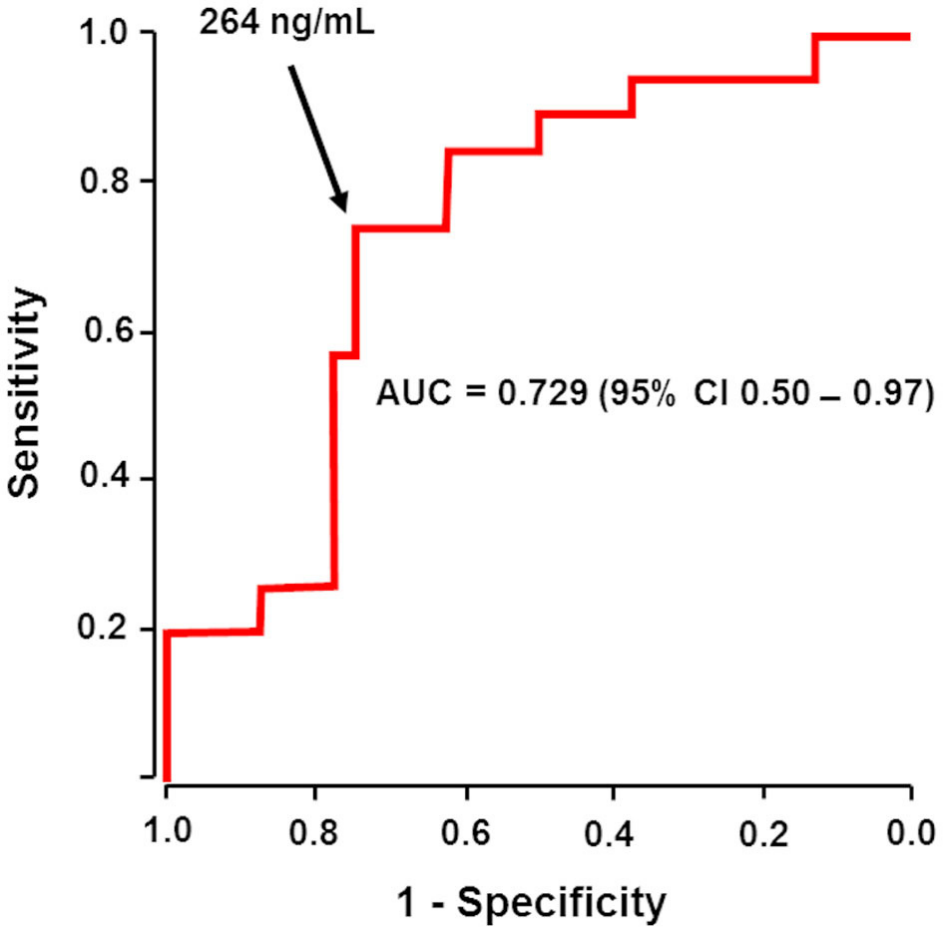
Initial plasma dabigatran could predict hemostatic effectiveness after reversal. The receiver operating characteristic (ROC) area under the curve (AUC) of the association between baseline plasma dabigatran level and hemostatic efficacy (effective or ineffective) in evaluable patients with major bleeding receiving idarucizumab. Arrow shows that a dabigatran level higher than 264 ng/mL before reversal was a predictor of ineffective hemostasis, with an area under the ROC curve of 0.729 (95% CI 0.50 − 0.97).

Then the relationship between the dabigatran level before reversal and adjudicated hemostatic efficacy was evaluated by ROC curves. Overall, there was a significant relationship between hemostatic efficacy and baseline dabigatran level. For patients with major bleeding, the highest likelihood ratio corresponded to a plasma dabigatran ≥264 ng/mL, which was a predictor of ineffective hemostasis, with an area under the ROC curve of 0.729 (95% CI 0.50–0.97, Figure 1). This value had a sensitivity of 82.4% (95% CI 55.0–92.0), a specificity of 75.0% (95% CI 49.0– 90.0), and a negative predictive value of 66.6 (95% CI 34.0–82.0) and a positive predictive value of 87.5 (95% CI 62.0–97.0).

Next, to assess the relevance of this cutoff according to renal impairment, we used a multivariable logistic regression model evaluating dabigatran level ≥264 ng/mL and CrCl <30 mL/min for hemostatic effectiveness outcomes. Adjustment to CrCl was chosen as the Cockcroft and Gault formula based on age, sex, and weight of the patients. Indeed, when a logistic regression model used the cutoff of 30 mL/min for CrCl, the association between dabigatran level <264 ng/mL and hemostatic effectiveness outcome remained significant [OR, 10.75 (95% CI 1.40–229.83), *p* = 0.046].

### Patient Characteristics According to Baseline Dabigatran Level Cutoff of 264 ng/mL

**Figure 2:**
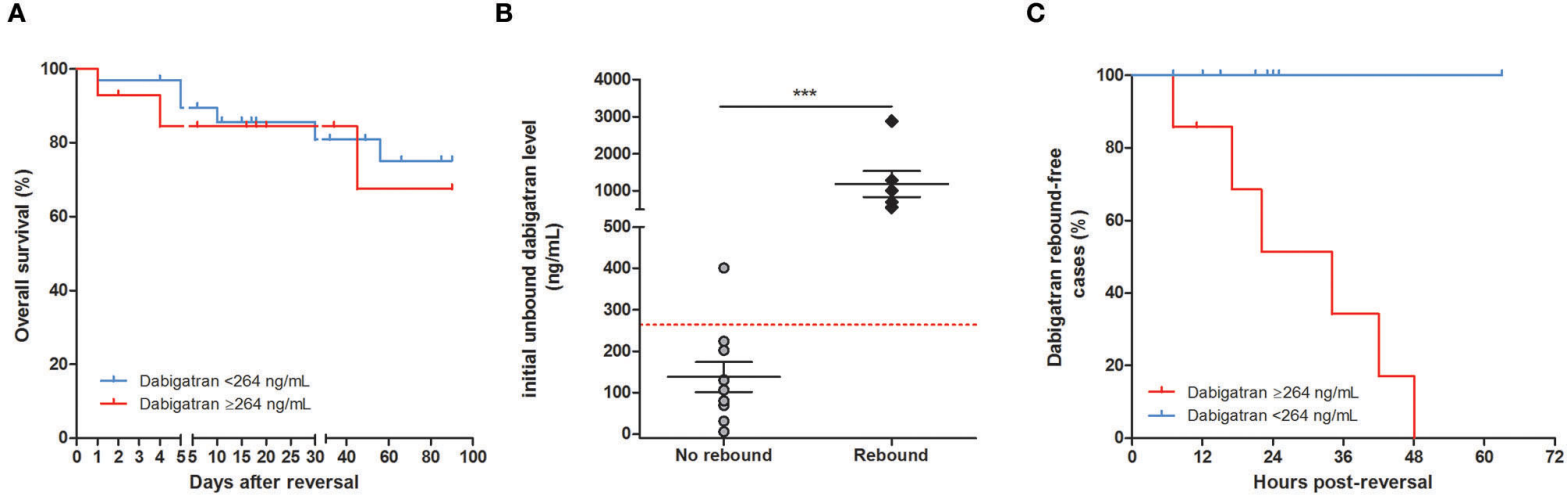
Initial plasma dabigatran level and biological or clinical outcomes. **(A)** Kaplan–Meier 90-day survival curve patients after idarucizumab administration according to baseline plasma dabigatran cutoff of 264 ng/mL. Percent survival rate was stratified by dabigatran plasma level at baseline < or ≥264 ng/mL for (*n* = 33 and 14, respectively) and did not significantly differ between groups (*p* = 0.36). **(B)** Initial plasma dabigatran concentrations in patients according to the presence or absence of dabigatran rebound after idarucizumab. Among the 87 patients who had a laboratory test prior to idarucizumab injection, only 16 had plasma dabigatran determination both at baseline and during follow-up. At baseline, patients with no rebound had a significant lower mean of dabigatran level (138 ng/mL ± 114, *p* = 0.0002) than patients with rebound (1,189 ng/mL ± 869). The dashed red line is a reference line indicating the predefined cutoff of 264 ng/mL. **(C)** Proportion of cases of dabigatran rebound after reversal according to the cutoff of 264 ng/mL before reversal. No dabigatran rebound was observed after reversal in patients with dabigatran <264 ng/mL at baseline (*n* = 9, blue bar). Among patients with dabigatran ≥264 ng/mL at baseline, rebound was observed in six patients within a median time of 34 h post-reversal (red bar).

The 264 ng/mL cutoff as determined above was used to compare baseline characteristics of the 47 patients of the entire cohort who had a specific dabigatran plasma measurement before reversal (Table 4). At admission, patients with a dabigatran level ≥264 ng/mL had a significantly lower CrCl (31.8 ± 20.8 vs. 50.7 mL/min ± 25.1, *p* = 0.019) and a significantly lower hemoglobin level (90.4 ± 22.4 vs. 111.3 g/L ± 30.1, *p* = 0.028). However, no difference in overall survival at 90 days was found between patients with plasma dabigatran < or ≥ 264 ng/mL at admission (Figure 2A, *p* = 0.48).

**Table 4:** Clinical and biological characteristics at admission of evaluable patients with or without baseline dabigatran ≥264 ng/mL.

**Patient characteristics**	**Dabigatran <264 ng/mL** **(*n* = 26)**	**Dabigatran ≥ 264 ng/mL** **(*n* = 21)**	***p*-value**
Age–year—mean (±SD)	80.4 (±12.5)	83.6 (±8.8)	0.39
Male sex—*n* (%)	23 (69.7)	6 (42.9)	0.16
CrCl–mL/ min—mean (±SD)	50.7 (±25.1)	31.8 (±20.8)	**0.019**
BMI–kg/m^2^–mean (±SD)	25.2 (±2.8)	27.7 (±6.5)	0.16
CHA_2_DS_2_-VASc score—mean (±SD)	3.4 (±1.4)	4.2 (±1.9)	0.16
Hemoglobin g/L—mean (±SD)	111.3 (±30.1)	90.4 (±22.4)	**0.028**

*BMI, body mass index; CrCl, creatinine clearance. Bold values are corresponding to statistical differences with p−value < 0.005*.

### Plasma Dabigatran Rebound After Reversal

In RE-VERSE AD trial (1), efficacy of idarucizumab was defined as the capacity to reverse the dabigatran anticoagulant effect within 4 h after its administration in dabigatran-treated patients. In our study, only one case of incomplete reversal was observed in patient #87 who had an initial dabigatran concentration of 2,881 ng/mL. Dabigatran level dropped to 73 ng/mL within 30 min after reversal and re-increased to 870 ng/mL 6 h later. We noticed eight (42.1%) reappearances of plasma dabigatran (mean 273 ng/m ± 288) between 7 and 48 h after idarucizumab infusion in 19 evaluable patients. When considering the 16 patients whose dabigatran plasma was measured both at baseline and during follow-up, patients experiencing dabigatran rebound had a significantly higher mean dabigatran concentration at admission (1,189 ± 869 vs. 138 ng/mL ± 114, *p* = 0.0002, Figure 2B).

Among the 16 patients with repeated measurements, seven patients had an initial plasma concentration ≥264 ng/mL and 9 patients <264 ng/mL. No plasma dabigatran rebound was observed after reversal in patients with dabigatran <264 ng/mL at baseline. In contrast, six (85.7%) patients with dabigatran ≥264 ng/mL at baseline had rebound within a mean time of 34 h post-reversal (Figure 2C). Patients with dabigatran rebound had a significantly higher mean CHA_2_DS_2−_VASc score (4.75 ± 1.9 vs. 3.10 ± 1.2, *p* = 0.039). Age, body mass index, plasma creatinine level, and CrCl were not associated with plasma dabigatran rebound (*p* > 0.05 for each, Table 5).

**Table 5:** Clinical and biological characteristics at admission of evaluable patients with or without dabigatran rebound after dabigatran reversal.

**Patient characteristics**	**No rebound** **(*n* = 11)**	**Rebound** **(*n* = 8)**	***p*-Value**
Age–year—mean (±SD)	80.73 (±10.4)	74.75 (±23.9)	0.46
Male sex—*n* (%)	9 (81.8)	3 (37.5)	0.13
CHA_2_DS_2_-VASc score–mean (±SD)	3.10 (±1.2)	4.75 (±1.9)	**0.039**
BMI–kg/m^2^–mean (±SD)	28.2 (±3.5)	27.0 (±7.7)	0.76
CrCl–mL/ min—mean (±SD)	62.2 (±34.5)	28.5 (±25.3)	0.054
Hemoglobin g/L—mean (±SD)	113.8 (±28.7)	117.7 (±48.9)	0.83
Dabigatran–ng/mL—mean (±SD)	138.0 (±115)	1,189.33 (±870)	**0.0002**

*BMI, body mass index; CrCl, creatinine clearance. Bold values are corresponding to statistical differences with p−value < 0.005*.

It should be noted that patient #57 with baseline dabigatran at 401 ng/mL did not experience dabigatran rebound; however, it should be specified that dabigatran monitoring was performed only during the first 12 h post-reversal.

## Discussion

**Figure 3:**
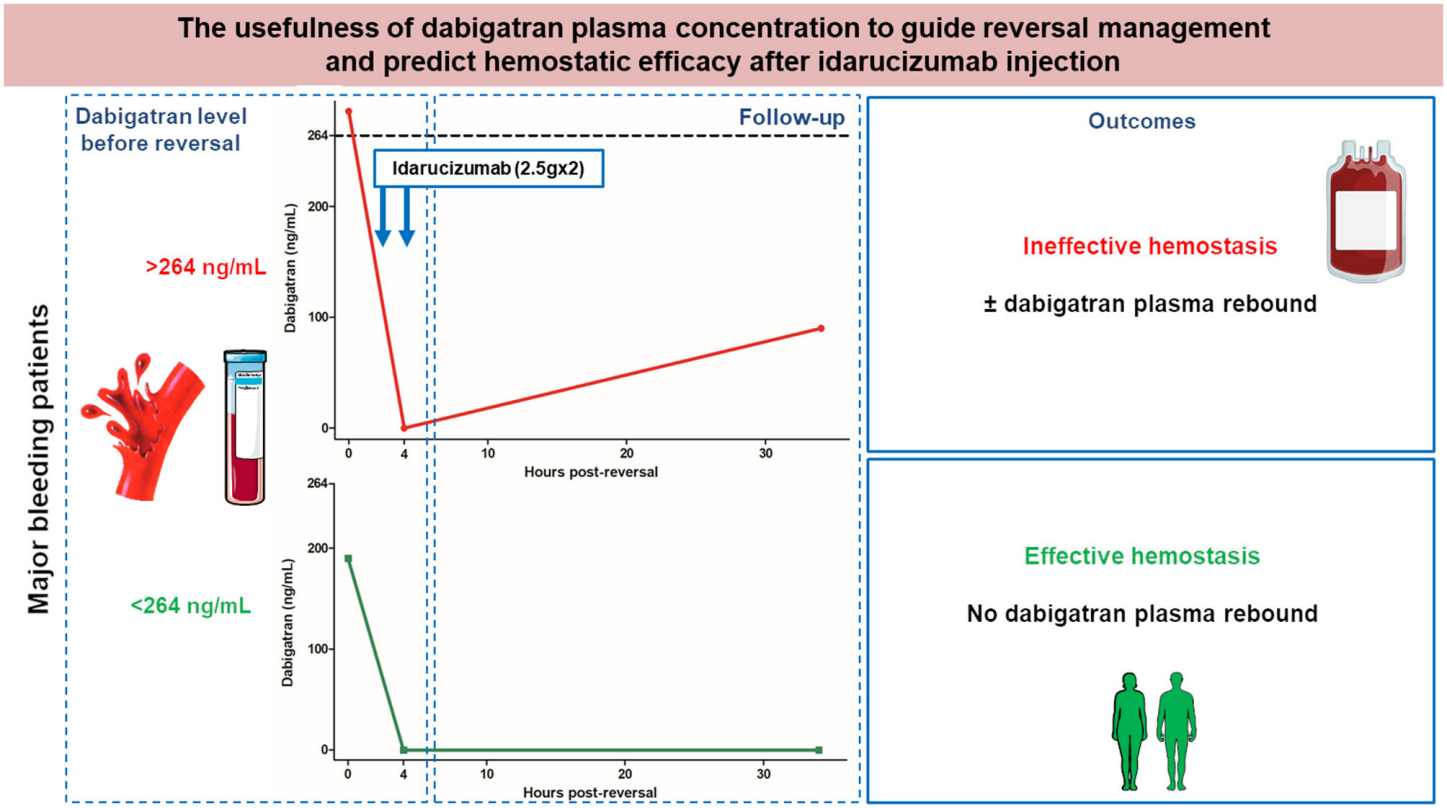
The usefulness of dabigatran plasma concentration to guide reversal management and predict hemostatic efficacy after idarucizumab injection.

This study reports on the biological and clinical experience of dabigatran reversal with idarucizumab in 21 French centers. The main finding of this real-world based cohort study is that initial plasma level of dabigatran before reversal allows to predict both clinical hemostatic effectiveness and risk of plasma dabigatran rebound. In addition, we showed that patients with ineffective hemostasis after reversal had less favorable outcomes and a trend to increased mortality. We identified patients who could not achieve effective hemostasis and/or might have significant circulating dabigatran rebound after reversal and a potential risk of non-surgical or surgical bleeding related to this rebound. The proposed dabigatran cutoff of 264 ng/mL could help in managing patients with uncontrolled bleeding after a first idarucizumab infusion, or patients who require a powerful neutralization for example in case of intravenous thrombolysis for stroke. Levy et al. (23) showed in the RE-VERSE AD trial that the median time from the first vial of idarucizumab to surgery or procedures was <2 h in all groups except neurosurgery, where it was 3.3 h. Idarucizumab should indeed be injected without awaiting the laboratory results, but dabigatran level at admission could help in anticipating the need for subsequent injections and influence follow-up after reversal (Figure 3).

For patients presenting major bleeding according to the ISTH criteria (14), treatment with idarucizumab was considered effective in 77.2% of them. Hemostatic effectiveness was similar to that reported in a large Dutch cohort (67%) (4). Among the entire cohort, 58.2% of evaluable patients received blood products or additional pro-hemostatic agents. The incidence of thrombotic events (3.4%) was similar to that reported in the RE-VERSE AD trial (4.8%), and none of these patients had any additional use of PCC or any other pro-hemostatic agents. Regarding thrombotic events, beyond the abrupt interruption of anticoagulation, there is also the risk resulting from immobilization, inflammation, surgery, and the overall context of intensive care, which makes the determination of the specific role of idarucizumab challenging in such events. We analyzed the use of idarucizumab in a real-world setting without questioning the indication for dabigatran reversal criteria followed by each center.

DOACs do not require biological monitoring since they have predictable pharmacokinetics and therapeutic effects (10). Idarucizumab is recommended at a single dose of 5 g (2.5 g × 2), calculated to reverse in a stoichiometric way the total body load of dabigatran that was associated with the 99th percentile of the dabigatran levels measured in the phase III study RE-LY. However, a single 5 g dose of idarucizumab would not be sufficient to completely reverse the effect of dabigatran in all cases. In RE-VERSE AD (1), the authors observed a plasma dabigatran rebound within 12 h post-reversal in 23% of the cases, and this rebound was associated with recurrent or continued bleeding in 9% of them, leading the investigators to consider a second administration of idarucizumab. In the present study, four (4.6%) patients received two or more injections of idarucizumab. Among them, three had a dabigatran initial concentration above 264 ng/mL (see Supplementary Table 4), similar to what was observed in the seven patients of the RE-VERSE AD trial (1) with re-bleeding and/or post-operative bleeding and who had a second injection of idarucizumab. In the RE-VECTO surveillance program (24), five bleeding patients required a second idarucizumab infusion for re-bleeding/prolonged coagulation test (*n* = 4) or for an additional urgent intervention (*n* = 1) suggesting insufficient hemostatic effectiveness after the first 5 g of infusion. As we previously showed (3, 25), the present study reports that bleeding patients had higher dabigatran levels at baseline compared to REVERSE-AD (337 vs. 110 ng/mL).

When assessing clinical hemostatic efficacy of idarucizumab treatment according to dabigatran concentration at admission, we determined a cutoff of 264 ng/mL as a predictor of efficacy. Moreover, patients with dabigatran rebound after reversal had dabigatran level at baseline above 264 ng/mL, a value close to the 200 ng/mL cutoff we previously suggested elsewhere (3). Dabigatran rebound is thought to occur as a result of redistribution in plasma of unbound dabigatran from the peripheral tissues to the intravascular compartment over time after idarucizumab clearance, in response to the concentration gradient occurring after neutralization as previously observed in healthy volunteers (26). Dabigatran and idarucizumab are both cleared by the kidneys. Recently, Eikelboom et al. (27) evaluated the impact of renal impairment on the effect of dabigatran reversal by idarucizumab in RE-VERSE AD. They showed that dabigatran rebound within 12–24 h was more common in patients with renal impairment, whereas time to bleeding cessation and extent of hemostasis during procedures were independent of renal function. Our results are similar, as hemostatic effectiveness did not correlate with renal function. It is important to consider that renal function is not a static measure, and in both RE-VERSE AD and the present study, it was only assessed before dabigatran reversal. Moreover, we found no significant difference between plasma creatinine level and CrCl in patients with or without plasma dabigatran rebound after reversal as previously described by our team (3). Furthermore, Glund et al. (28) showed that subjects with mild or moderate renal impairment had an increased exposure, a decreased clearance, and a prolonged half-life of idarucizumab compared to healthy subjects. Hence, according to our results, hemostatic efficacy would be driven by a balance between dabigatran extravascular accumulation/redistribution and the neutralization capacity of circulating idarucizumab along time.

There are several limitations to consider with respect to our study. First, the retrospective design of the present study based on reported cases could limit data quality. Large-scale registers of the use of idarucizumab are still needed and should be implemented by dabigatran monitoring at baseline and during reversal to analyze the clinical relevance of dabigatran rebound. Second, as in the RE-VERSE AD study, the lack of a control group and the small number of patients may limit the strength of the conclusions.

In summary, our study shows that dabigatran level at admission is a major tool to predict clinical hemostasis upon idarucizumab reversal as well as plasma dabigatran rebound. Using a cutoff of 264 ng/mL, plasma dabigatran before reversal in patients with major bleeding allows indeed to predict hemostatic ineffectiveness, dabigatran rebound, and outcomes after reversal. Dabigatran level before reversal is not intended to “treat or not-treat” but to select patients that might need a specific follow-up after reversal with idarucizumab. The proposed cutoff should be included in further prospective studies to be validated.

## Data Availability Statement

The raw data supporting the conclusions of this article will be made available by the authors, without undue reservation.

## Ethics Statement

The studies involving human participants were reviewed and approved by the institutional review board of each center and anonymous data collection was declared to the appropriate authorities (Authorization protocol number: CNIL-1922081). Written informed consent for participation was not required for this study in accordance with the national legislation and the institutional requirements.

## Author Contributions

NG, RC, ALL, and DS conceived and supervised the study. NG, RC, PB, ALL, and DS analyzed and interpreted the data. RC analyzed the data and supervised the statistical analysis. NG, RC, PB, PG, and DS wrote the manuscript. All authors managed and included patient cases. All authors revised the manuscript and approved the final version.

## Conflict of Interest

NG discloses consulting fees by Boehringer Ingelheim, Bayer, Bristol-Myers Squibb/Pfizer and LEO Pharma. RC reports consulting fees from Aspen. A-CM discloses consulting fees from Bayer and Boehringer Ingelheim, and consulting fees and grant from Bristol-Myers-Squibb/Pfizer. ALL discloses consulting fees by Boehringer Ingelheim and Bayer. LB reports personal fees and non-financial support from Aspen, Actelion, Bayer, LEO-pharma, Bristol-Myers Squibb/Pfizer, and MSD; non-financial support from Daiichi; and grants and personal fees from Sanofi outside the submitted work. DS declares consulting fees from Bayer, Bristol-Myers Squibb/Pfizer, Boehringer Ingelheim, Aspen, and LEO-Pharma. The remaining authors declare that the research was conducted in the absence of any commercial or financial relationships that could be construed as a potential conflict of interest.
