# Supplementary material for: Dabigatran Level Before Reversal Can Predict Hemostatic Effectiveness of Idarucizumab in a Real-World Setting
Source: Front Med (Lausanne). 2020 Dec 16;7:599626. doi: 10.3389/fmed.2020.599626 (PMC7772865; doi:10.3389/fmed.2020.599626)
Supplement: Supplementary file 1 [file Data_Sheet_1.docx]

Supplementary Material

# Supplementary Figures and Tables

## Supplementary Tables

**Supplemental Table 1. Patient characteristics at admission in the bleeding group and outcomes after dabigatran reversal**

M for male; F for female; BID for twice a day; NA for non-available; PRBCs for packed red blood cells, FFP for fresh frozen plasma, PCC for prothrombin complex concentrate, TxA for tranexamic acid, CRNM for clinically relevant non major bleeding, DIC for disseminated intravascular coagulation.

*Calculated with Cockcroft and Gault formula.
**Patients #37, #38 and #39 had specific measurement of total dabigatran level with LC/MS.

**Supplemental Table 2. Patient characteristics at admission in the urgent procedure group and outcomes after dabigatran reversal**

M for male; F for female; BID for twice a day; NA for non-available; PRBCs for packed red blood cells, FFP for fresh frozen plasma, PCC for prothrombin complex concentrate, TxA for tranexamic acid.
*Calculated with Cockcroft and Gault formula

**Supplemental Table 3. Characteristics of the two patients with overdose at admission and outcomes after dabigatran reversal**

F for female; BID for twice a day.

*Calculated with Cockcroft and Gault formula **without bleeding

| **Pt** | **Age (year)** | **Sex** | **Dose of dabigatran** | **Index event** | **Hemoglobin (g/L)** | **Creatinine clearance (mL/min)*** | **Baseline level of unbound dabigatran (ng/mL)** | **Additional blood products or hemostatic agents used** | **Outcomes** |
| --- | --- | --- | --- | --- | --- | --- | --- | --- | --- |
| **86** | 72 | F | 150 BID | Overdose** | 120 | 10.8 | 1014 | - | Favorable |
| **87** | 79 | F | 150 BID | Overdose** | 80 | 5.0 | 2881 | - | Favorable |

**Supplemental Table 4. Characteristics of patients who received more than one dose of idarucizumab.**

M for male; F for female; BID for twice a day; NA for non-available;
*Calculated with Cockcroft and Gault formula
**without bleeding
*** This patient had a third injection of idarucizumab three days after the second injection due to high plasma rebound and a kidney biopsy was considered. No bleeding event was observed during hospitalization.

| **Pt** | **Age (year)** | **Sex** | **Dose of dabigatran** | **Index event** | **Creatinine clearance (mL/min)*** | **Baseline level of dabigatran (ng/mL)** | **Approximate time to additional dose** | **Reason for additional dose** |
| --- | --- | --- | --- | --- | --- | --- | --- | --- |
| **8** | 79 | F | 110 BID | Gastrointestinal bleeding | 31.7 | **702** | 12 hours | Plasma dabigatran rebound at 124 ng/mL before invasive procedure |
| **9** | 99 | F | 110 BID | Gastrointestinal bleeding | 12.9 | **1284** | 24 hours | Plasma dabigatran rebound at 420 ng/mL |
| **76** | 69 | M | 150 BID | Mechanical thrombectomy | 75.0 | NA | 2 hours | Bleeding transformation |
| **87** | 79 | F | 150 BID | Overdose** | 5.0 | **2881** | 48 hours | Plasma dabigatran rebound at 1250 ng/mL before invasive procedure*** |

**Supplemental Table 5. Additional blood products or hemostatic agents used**

PRBC for packed red blood cells; PCC for prothrombin complex concentrate; FFP for fresh frozen plasma.
*Patients #86 and #87 from received idarucizumab for dabigatran overdose without bleeding and did not receive additional blood products or pro-hemostatic agents.

| **Additional blood products or pro-hemostatic agents** | **Bleeding (N=61)** | **Urgent procedure (N=24)** | **All patients (N=87)*** |
| --- | --- | --- | --- |
| PRBCs - n (%) | 31 (50.8) | 8 (33.3) | 39 (44.8) |
| FFP - n (%) | 5 (8.2) | 5 (20.8) | 10 (11.5) |
| PCC - n (%) | 4 (6.6) | 2 (8.3) | 6 (6.9) |
| Platelets transfusion - n (%) | 1 (1.6) | 4 (16.6) | 5 (5.7) |
| Tranexamic acid - n (%) | 2 (3.3) | 1 (4.2) | 3 (3.4) |
| Fibrinogen - n (%) | - | 1 (4.2) | 1 (1.1) |
| Vitamin K - n (%) | 1 (1.6) | - | 1 (1.1) |
| Missing data - n (%) | 5 (8.2) | 3 (12.5) | 8 (9.2) |

**Supplemental Table 6. Antithrombotic therapy resumption during follow-up**

VKA for vitamin K antagonist; LWMH for low weight molecular heparin; UFH for unfractioned heparin.
*Patients #86 and #87 received idarucizumab for dabigatran overdose without bleeding.

| **Antithrombotic therapy resumption** | **Bleeding (N=61)** | **Urgent procedure (N=24)** | **Others* (N=2)** | **All patients (N=87)** |
| --- | --- | --- | --- | --- |
| None - n (%) | 32 (52.5) | 5 (20.8) | - | 37 (42.5) |
| Dabigatran - n (%) | 15 (24.6) | 12 (50) | 1 (50.0) | 28 (32.2) |
| VKA - n (%) | 3 (4.9) | 2 (8.3) | 1 (50.0) | 6 (6.9) |
| Apixaban - n (%) | 3 (4.9) | 1 (4.1) | - | 4 (4.6) |
| Rivaroxaban - n (%) | 1 (1.6) | - | - | 1 (1.1) |
| LWMH - n (%) | 2 (3.3) | - | - | 2 (2.3) |
| UFH - n (%) | 2 (3.3) | 2 (8.3) | - | 4 (4.6) |
| Antiplatelet therapy - n (%) | 1 (1.6) | 1 (4.1) | - | 2 (2.3) |
| Missing data - n (%) | 2 (3.3) | 1 (4.1) | - | 3 (3.4) |

**Supplemental Table 7. Cause of death of the 18 patients after idarucizumab administration.**

|  | **Bleeding (N=61)** | | | | **Urgent procedure (N=24)** | | | |
| --- | --- | --- | --- | --- | --- | --- | --- | --- |
| Timing of death | **5 days** | **30 days** | **90 days** | **Total** | **5 days** | **30 days** | **90 days** | **Total** |
|  |  |  |  |  |  |  |  |  |
| All cause of death - n (%) | 10 (16.4) | 2 (3.3) | 2 (3.3) | 14 (22.9) | 2 (8.3) | 1 (4.1) | 1 (4.1) | 4 (16.6) |
| Fatal bleeding | 5 (8.2) | - | 1 (1.6) | 6 (9.8) | 1 (4.1) | 1 (4.1) | - | 2 (8.3) |
| Re-bleeding | - | - | - | - |  | - | - |  |
| Severe infection | 1 (1.6) | 1 (1.6) | 1 (1.6) | 3 (4.9) | - | - | - | - |
| Multivisceral failure | 2 (3.3) | 1 (1.6) | - | 3 (4.9) | 1 (4.1) | - | 1 (4.1) | 2 (8.3) |
| Cardiac arrest | 1 (1.6) |  |  | 1 (1.6) |  |  |  |  |
| Disseminated intravascular coagulation | 1 (1.6) | - | - | 1 (1.6) | - | - | - | - |

**Supplemental Table 8. Characteristics of patients with plasma dabigatran rebound after reversal.**

M for male; F for female; BID for twice a day; NA for non-available;
*Calculated with Cockcroft and Gault formula
**without bleeding

| **Pt** | **Age (year)** | **Sex** | **Dose of dabigatran** | **Index event** | **Creatinine clearance (mL/min)*** | **Baseline level of dabigatran (ng/mL)** | **Level of dabigatran <12 after reversal  (ng/mL)** | **Level of dabigatran >12h after reversal (ng/mL)** | **Outcomes, causes of death** |
| --- | --- | --- | --- | --- | --- | --- | --- | --- | --- |
| **8** | 79 | F | 110 BID | Gastrointestinal bleeding | 31.7 | 702 | - | 124 | Favorable |
| **9** | 99 | F | 110 BID | Gastrointestinal bleeding | 12.9 | 1284 | <30 | 420 | Favorable |
| **22** | 93 | M | 110 BID | Gastrointestinal bleeding | 60.0 | 700 | <30 | 54 | Favorable |
| **32** | 82 | M | 110 BID | Intracranial bleeding | NA | NA | 53 | - | Death, cardiac arrest |
| **67** | 20 | F | 150 BID | Heart transplant | 66.9 | NA | <30 | 182 | Favorable |
| **80** | 74 | M | 150 BID | Urologic surgery | 12.6 | 555 | 54 | 142 | Favorable |
| **86** | 72 | F | 150 BID | Overdose** | 10.8 | 1014 | <30 | 118 | Favorable |
| **87** | 79 | F | 150 BID | Overdose** | 5.0 | 2881 | 870 | 1250 | Favorable |

## Supplementary Figures

**Supplemental figure 1. Kaplan–Meier 90-day survival curve after idarucizumab administration according to indication.** Percent survival rate was stratified by idarucizumab indication for bleeding (70.3%, n=61) or urgent procedure (83.3, n=24). Percent survival rate did not significantly differ between groups (p=0.92).

**
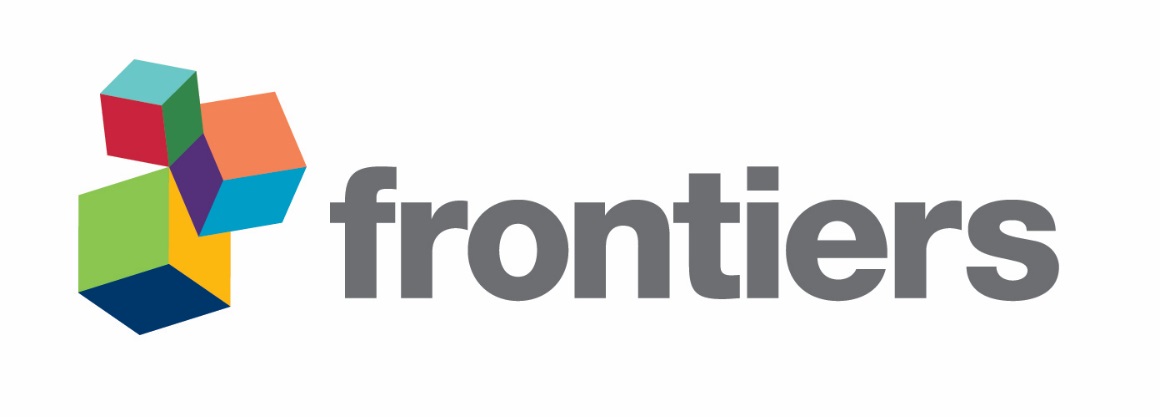
**
